# Effect of luteolin on oxidative stress and inflammation in the human osteoblast cell line hFOB1.19 in an inflammatory microenvironment

**DOI:** 10.1186/s40360-024-00764-4

**Published:** 2024-07-12

**Authors:** Zhengjun Peng, Wenyu Zhang, Hong Hong, Lu Liu

**Affiliations:** Operative Dentistry and Endodontics, Guanghua School of Stomatology, Guangdong Province Key Laboratory of Stomatology, Affiliated Stomatological Hospital, Sun Yat-Sen University, 56 Lingyuan Xi Rd, Guangzhou, 510055 Guangdong China

**Keywords:** Luteolin, Chronic apical periodontitis, Inflammation, Oxidative stress, NF-κB signalling pathway

## Abstract

**Background:**

Periapical lesions are characterized by periapical inflammation and damage to periapical tissues and eventually lead to bone resorption and even tooth loss. H_2_O_2_ is widely used in root canal therapy for patients with periapical inflammation. Luteolin possesses high anti-inflammatory, antioxidant, and anticancer potential. However, the underlying mechanism of the efficacy of H_2_O_2_ and luteolin on oxidative stress and inflammatory tissue has not been previously addressed. We aimed to investigate the anti-inflammatory and antioxidative effects of luteolin on H_2_O_2_-induced cellular oxidative inflammation.

**Methods:**

After human osteoblasts (hFOB1.19) were treated with lipopolysaccharide (LPS), luteolin, or H_2_O_2_, cell proliferation was analysed by using a cell counting kit-8 (CCK-8), cell apoptosis was measured by using flow cytometry, the production of reactive oxygen species (ROS) was evaluated by using an oxidation-sensitive probe DCFH-DA ROS assay kit, and the expression of genes and proteins was detected by using reverse transcription quantitative polymerase chain reaction (RT‒qPCR), Western blotting, and enzyme-linked immunosorbent assay (ELISA).

**Results:**

We demonstrated that inflammation is closely related to oxidative stress and that the oxidative stress level in the inflammatory environment is increased. Luteolin inhibited the H_2_O_2_-induced increase in the expression of interleukin-6 (IL-6), interleukin-8 (IL-8) and tumour necrosis factor α (TNF-α) and significantly repressed the H_2_O_2_-induced increase in ROS, as well as markedly strengthened superoxide dismutase (SOD) activity in hFOB1.19 cells. Moreover, we detected that luteolin may inhibit H_2_O_2_-induced hFOB1.19 cell injury by suppressing the NF-κB pathway.

**Conclusion:**

We elucidated that luteolin protected human osteoblasts (hFOB1.19) from H_2_O_2_-induced cell injury and inhibited the production of proinflammatory cytokines by suppressing the NF-κB signalling pathway. Our findings provide a potential drug for treating H_2_O_2_-induced periodontitis and cell injury.

## Background

Chronic apical periodontitis (AP), which commonly originates from dental pulp infection caused by gram-negative anaerobic bacteria and their products, is a local nonspecific inflammation that occurs in the apical region [1]. The host attempts to limit the further spread of infection through its immune inflammatory response [2]. Clinically, the loss of apical tissues, including the periodontal ligament and radicular cementum, and injury to the alveolar bone can occur. This osteolytic lesion has been characterized by chronic periapical inflammation [3]. Histologically, the apical tissue is replaced by inflammatory tissue, and the apical lesion is composed of granulation tissue, which may progress to form a radicular cyst, which is a pathological cavity lining the squamous epithelium [4]. The entire process can be regarded as being a continuous stage of the disease [1].

Inflammation is closely related to oxidant stress [5]. Inflammatory cells produce reactive oxygen species (ROS), which may lead to increased oxidative stress in inflammatory lesions [5]. It has been reported that inflammation regulates the Keap1/Nrf2 pathway, which plays a crucial role in oxidative stress by increasing miR-200 [6]. Correspondingly, ROS enhance the expression of proinflammatory genes by stimulating the intracellular signalling cascade [7]. ROS can promote the progression of inflammation through acetyl-p53 [8]. Therefore, the close interaction between oxidative stress and inflammation forms a vicious cycle to promote the pathogenesis of the disease.

Root canal therapy through root canal preparation, irrigation and filling is currently the most common treatment for chronic apical periodontitis of the teeth [9]. Although sodium hypochlorite has been more commonly used as a root canal irrigation solution in large dental institutes, H_2_O_2_ is still widely used in primary medical units [10]. In recent years, studies have indicated that H_2_O_2_ has poor anti-inflammatory and sterilization effects on chronic periapical periodontitis [11]. After treatment, the apical bone defect may continue or recur, which results in apical surgery or tooth extraction as the final treatment. However, the mechanism underlying H_2_O_2_-mediated regulation of inflammation is unknown.

Luteolin (3′,4′,5,7-tetrahydroxyflavone), which is a natural source of flavonoids in many plants, is a compound with rich biological activities [12]. Luteolin has a variety of pharmacological activities, such as anti-inflammatory, anti-allergic, antioxidative, anticancer, antidiabetic, and antiviral effects [13, 14]. Our previous studies have reported that luteolin can increase cell growth, inhibit apoptosis, maintain pluripotency through activation of the Oct-4/Sox2/c-Myc axis, and repress lineage-specific differentiation [15]. Our previous data showed that luteolin may be an efficient tissue-engineering bioflavonoid. In addition, several studies have demonstrated the inhibitory effect of luteolin on inflammation in multiple diseases [16]. For example, luteolin can ameliorate inflammation in rats with allergic rhinitis [17]; moreover, luteolin can alleviate inflammation in rats with ulcerative colitis [18], as well as weaken inflammation after cerebral ischaemia‒reperfusion in rats via PPARγ [19]. Furthermore, luteolin can attenuate oxidative stress and inflammation in chronic obstructive pulmonary disease [20]. However, the underlying role and mechanism of luteolin in inflammatory osteoblasts are still unclear.

In the present study, we explored the role of luteolin in oxidative stress and inflammation in hFOB1.19 cells in inflammatory microenvironments. Our findings provide a potential drug for treating H_2_O_2_-induced periodontitis and osteoblast injury.

## Methods

### Cell culture and establishment of an inflammatory cell model in vitro

Cryopreservation tubes containing the human osteoblast cell line hFOB1.19 were placed in a 37 °C water bath and shaken constantly until the liquid was heated and dissolved. The cells were transferred to a 15 ml centrifuge tube containing at least 10 ml of MEM (MEM + 10% foetal bovine serum + 100 U/ml penicillin + 100 U/ml streptomycin) and centrifuged for 5 min at 800 rpm. Subsequently, the cells were resuspended in 3 mL of culture medium and cultured in a 5% CO_2_ incubator at 37 °C.

An inflammatory cell model was constructed by using hFOB1.19 cells, which were induced with lipopolysaccharide (LPS; 10 µg/mL, Sigma, Cat No: L4391). After stimulation for 0, 6, 12, 24, 48, 72, or 96 h, the cells were collected and subjected to ELISA analysis. The inflammatory indices C-reactive protein (CRP) and interleukin-2 (IL-2), as well as the oxidative stress index superoxide dismutase (SOD), were detected and analysed in a human inflammatory osteoblast model.

hFOB1.19 cells were then randomly divided into five groups: the control group, inflammatory model group, H_2_O_2_ treatment group (the inflammatory model cells were treated with 200 µM/ml H_2_O_2_), luteolin treatment group (the inflammatory model cells were treated with 10 µmol/L luteolin [MedChemExpress, Monmouth Junction, NJ, Cat NO. 491-70-3]), and luteolin combined with H_2_O_2_ treatment group (the inflammatory model cells were treated with 200 µM/ml H_2_O_2_ and 10 µmol/L luteolin).

### Cell proliferation assays

Cell proliferation was evaluated by using a Cell Counting Kit-8 (CCK-8, Beyotime, Shanghai, China, Cat No: C0039) assay according to the manufacturer’s protocol. Approximately 1500 cells were seeded into 96-well plates and cultured for 0, 1, 3, or 5 days. CCK-8 solution and medium (1:10) were added, and the cells were incubated at 37 °C for 2 h. The absorbance was detected at 450 nm by using a microplate reader (Bio-Rad 680, Bio-Rad, Hercules, CA, USA).

### Flow cytometry for cell cycle and apoptosis analysis

For apoptosis, cell apoptosis was determined by using an annexin V-FITC/PI apoptosis detection kit (Nanjing KeyGen Biotech Co., Ltd., Cat. No. KGA106). The collected hFOB1.19 cells (1 × 10^5^ cells/well) were stained with annexin V-FITC (5 µL) and PI (5 µL) in the dark for 30 min. The apoptosis rate was analysed by using flow cytometry (FACSCanto II, BD Biosciences). UL (AnnexinV-FITC)^−^/PI^+^ represents necrotic cells; UR (AnnexinV-FITC)^+^/PI^+^ represents late apoptotic cells; LR (AnnexinV-FITC)^+^/PI^−^ represents early apoptotic cells; and LL (AnnexinV-FITC)^−^/PI^−^ represents viable cells. The percentage of apoptotic cells in the UR and LR quadrants was calculated as the sum of the percentages. For the cell cycle, the treated hFOB1.19 cells were fixed with 75% ethanol for 24 h. After centrifugation and washing, the cells were resuspended in 50 µg/ml propidium iodide (PI; BD Biosciences, Franklin Lakes, NJ, USA) and 100 µg/ml RNase (Takara Bio, Inc.) for 30 min. Afterwards, the cell cycle distribution was confirmed by using a flow cytometer.

### 2,2′-Azino-bis (3-ethylbenzothiazoline-6-sulfonic acid) (ABTS) free radical scavenging assay

The antioxidant capacity of the hFOB1.19 cells after 3 days of treatment was measured by using an ABTS free radical scavenging assay kit (Acmec, Cat No: AC10744-100T/48S) and a microplate reader based on the manufacturer’s instructions.

### Beta-galactosidase staining for cell senescence

Cell senescence was evaluated by using a β-galactosidase staining kit (Sigma‒Aldrich, Cat No: KAA002). hFOB1.19 cells were preincubated at a density of 5 × 10^5^ cells/well in a 6-well plate for 24 h, washed with PBS and fixed with paraformaldehyde. Subsequently, the cells were incubated with X-gal chromogenic substrate at pH 6.0 at 37 °C overnight according to the manufacturer’s instructions. The cell morphology was observed, and images were captured under an inverted light microscope.

### DCFH-DA ROS assays

The production of ROS in each group was evaluated by using an oxidation-sensitive probe DCFH-DA ROS assay kit (Sigma, Merck KGaA, Cat No: D6883). The cells in each group were preincubated for 24 h, and the cells were then incubated at 37 °C for 20 min and mixed with DCFH-DA solution (10 µmol/L). ROSUP was added to the positive control group as a positive control. The oxidative formation of DCFH-DA via intracellular ROS was evaluated by using a flow cytometer.

### Reverse transcription quantitative polymerase chain reaction (RT‒qPCR)

Total RNA was extracted by using TRIzol reagent, and cDNA was synthesized by using a Bestar qPCR RT Kit (DBI Bioscience, Cat No: 2220). RT‒qPCR was performed on an Agilent Stratagene Mx3000P RT‒qPCR machine (Agilent Stratagene, Santa Clara, CA, USA) with DBI Bestar^®^ SybrGreen qPCR Master Mix (DBI Bioscience, Cat No: DBI-2043). The expression of target genes was normalized to the expression of GAPDH and analysed via the 2^−△△ct^ method. The utilized primer pairs are shown in Table 1.

**Table 1:** Primers used for RT‒qPCR

Gene name	Forward primer	Reverse primer
IL-6	AGTCCTGATCCAGTTCCTGC	CTACATTTGCCGAAGAGCCC
IL-8	ACTGAGAGTGATTGAGAGTGGAC	AACCCTCTGCACCCAGTTTTC
TNF-α	GAGGCCAAGCCCTGGTATG	CGGGCCGATTGATCTCAGC
GAPDH	TGTTCGTCATGGGTGTGAAC	ATGGCATGGACTGTGGTCAT

### Enzyme-linked immunosorbent assay (ELISA)

The concentrations of TNF-α, IL-8, IL-6 and SOD were monitored by using TNF-α ELISA kits (R&D Systems, Inc., Minneapolis, MN, USA; Cat No: DTA00D), IL-8 ELISA kits (R&D Systems, Cat No: D8000C), IL-6 ELISA kits (R&D Systems, Cat No: D6050B), and SOD ELISA kits (R&D Systems, Cat No: DYC3419-2), respectively. The optical density (OD) at 450 nm was measured by using a microplate reader.

### Western blot analysis

The protein expression of IL-6, IL-8, TNF-α, p-p65, p65, lκB-α, and p-lκB-α was estimated via Western blotting. Total protein was extracted by using a radioimmunoprecipitation assay (RIPA, Beyotime, Shanghai, China, Cat No: P0013B), and the bicinchoninic acid (BCA) (Beyotime, Shanghai, China) method was used to quantify the total protein concentration. The protein samples were first electrophoresed for 2 h, after which the total protein was transferred to polyvinylidene difluoride (PVDF) membranes (Millipore, Billerica, MA, USA). TBST containing 5% skim milk was used to block nonspecific antigens for 1 h following incubation with primary and secondary antibodies (Abcam, Cambridge, MA, USA). The utilized primary antibodies are listed in Table 2. The bands were subjected to enhanced chemiluminescence (ECL, Amersham Biosciences, Piscataway, NJ, USA, Cat No: RPN3001) and analysed by using Image Software (NIH, Bethesda, MD, USA).

**Table 2:** Primary antibodies used for Western blotting

Name	Product code	Manufacturer	Dilution
IL-6	66146-1-Ig	Proteintech	1: 1000
IL-8	27095-1-AP	Proteintech	1: 1500
TNF-α	17590-1-AP	Proteintech	1: 2000
p65	80979-1-RR	Proteintech	1: 3000
p-p65	ab76302	abcam	1: 1000
lκB-α	10268-1-AP	Proteintech	1: 2000
p-lκB-α	120,980	Novopro	1: 1000
Nrf2	ab137550	Abcam	1: 1000
GAPDH	ab9485	Abcam	1: 2000

### Statistical analysis

Each experiment was repeated at least three times. All of the data are presented as the means ± standard deviations and were analysed by using SPSS 20.0 software. The Bartlett test was used to determine whether our data were normally distributed. Two-sided ANOVA or t tests were used to assess differences in normally distributed data. A *P* value < 0.05 was considered to indicate a statistically significant difference.

## Results

### Inflammation increases the oxidative stress level of hFOB1.19 cells

**Fig. 1:**
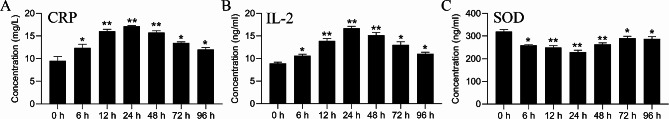
Effect of LPS on the protein expression of CRP, IL-2, and SOD in hFOB1.19 cells, as determined by ELISA. ELISA analysis demonstrated that after treatment with LPS, the concentrations of CRP and IL-2 gradually increased in hFOB1.19 cells and then decreased after 24 h of treatment (**A** and **B**). SOD activity gradually decreased in hFOB1.19 cells and then increased after 24 h of treatment (**C**). The data are expressed as the means ± SDs (**p < 0.01, *p < 0.05)

First, we established an inflammatory cell model by stimulating human osteoblast hFOB1.19 cells with LPS (10 µg/mL). Compared with the control, LPS treatment notably upregulated CRP and IL-2 levels in the supernatant of the culture system, although the expression levels tended to change and decrease after 24 h (Fig. 1A and B). Moreover, the expression level of SOD exhibited the opposite trend (Fig. 1C), whereby it initially decreased after treatment with LPS and then increased after 24 h. These findings suggested that inflammation may suppress the antioxidant system and increase oxidative stress levels in hFOB1.19 cells.

### Luteolin reduced H_2_O_2_-induced hFOB1.19 cell injury

**Fig. 2:**
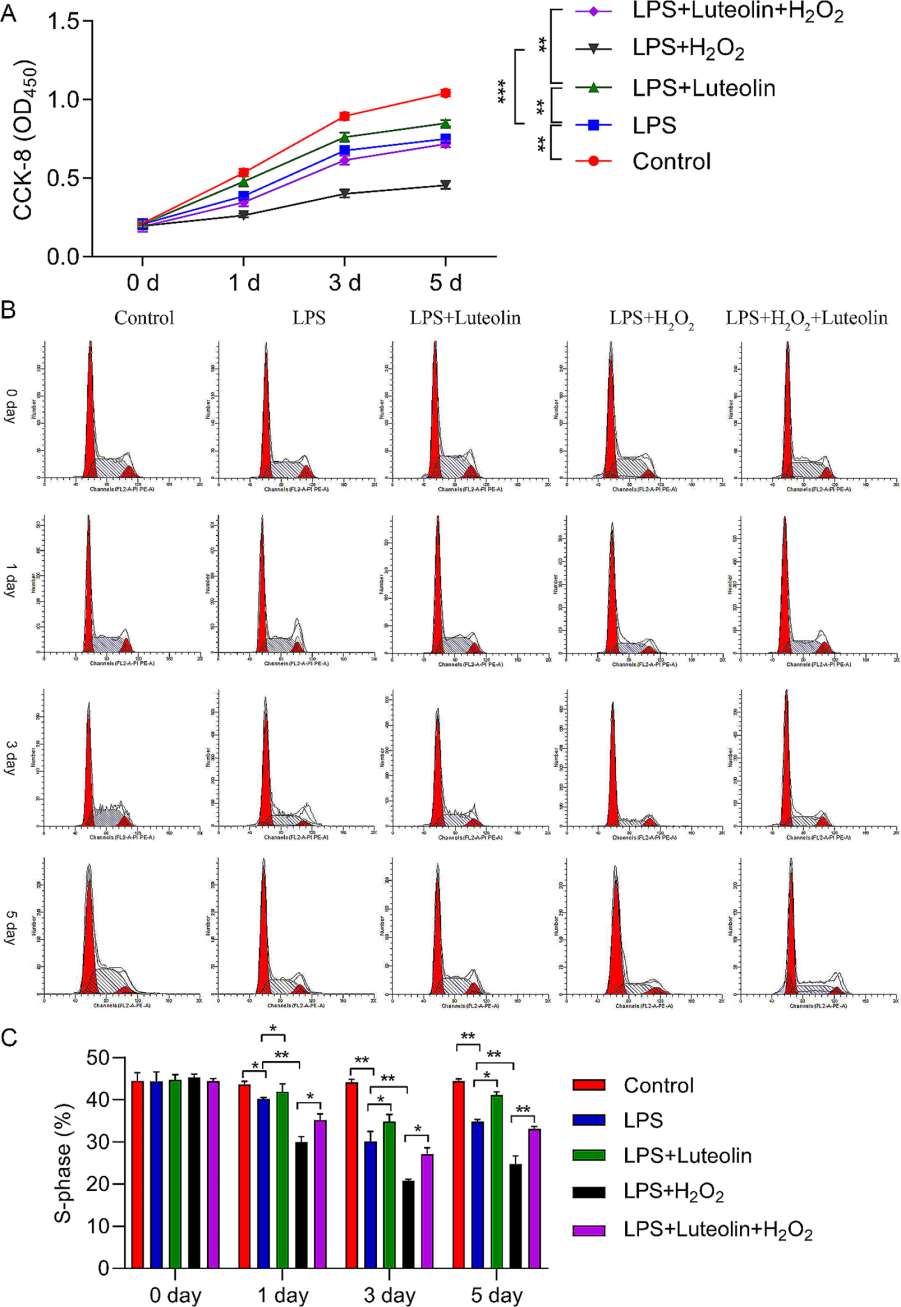
Effect of luteolin on the proliferation and cell cycle progression of hFOB1.19 cells. (**A**) The proliferation and cell cycle distribution of luteolin-treated hFOB1.19 cells were detected by using a CCK-8 assay. (**B**) The cell cycle distribution in each group was detected by using flow cytometry. (**C**) The proportion of S-phase cells was quantified. LPS induction reduced the proportion of S-phase hFOB1.19 cells after 1, 3, and 5 days, and this reduction was reversed by luteolin. With the combination of LPS and H_2_O_2_, the proportion of hFOB1.19 cells in the S phase was significantly reduced after 1, 3, and 5 days. The data are expressed as the means ± SDs (***p < 0.001, **p < 0.01, *p < 0.05)

To explore the potentially protective effect of luteolin in vitro, we treated hFOB1.19 cells with H_2_O_2._ As expected, LPS treatment notably suppressed cell viability and cell cycle distribution. Compared with LPS treatment, H_2_O_2_ treatment further markedly decreased the viability of hFOB1.19 cells. Furthermore, luteolin treatment increased the viability of hFOB1.19 cells, thus confirming the cytoprotective effect of luteolin treatment. Additionally, luteolin partly rescued the inhibitory effects of H_2_O_2_ on hFOB1.19 cells after LPS treatment (Fig. 2A).

At 1, 3, and 5 days, LPS treatment reduced the proportion of S-phase hFOB1.19 cells, which could also be reversed by luteolin; H_2_O_2_ treatment further enhanced the reduction in the proportion of S-phase hFOB1.19 cells mediated by LPS. Additionally, H_2_O_2_ treatment markedly decreased the proportion of S-phase hFOB1.19 cells, which were increased by luteolin treatment in LPS-induced hFOB1.19 cells, and luteolin treatment markedly increased the proportion of S-phase hFOB1.19 cells, which were reduced by H_2_O_2_ treatment in LPS-induced hFOB1.19 cells (Fig. 2B).

**Fig. 3:**
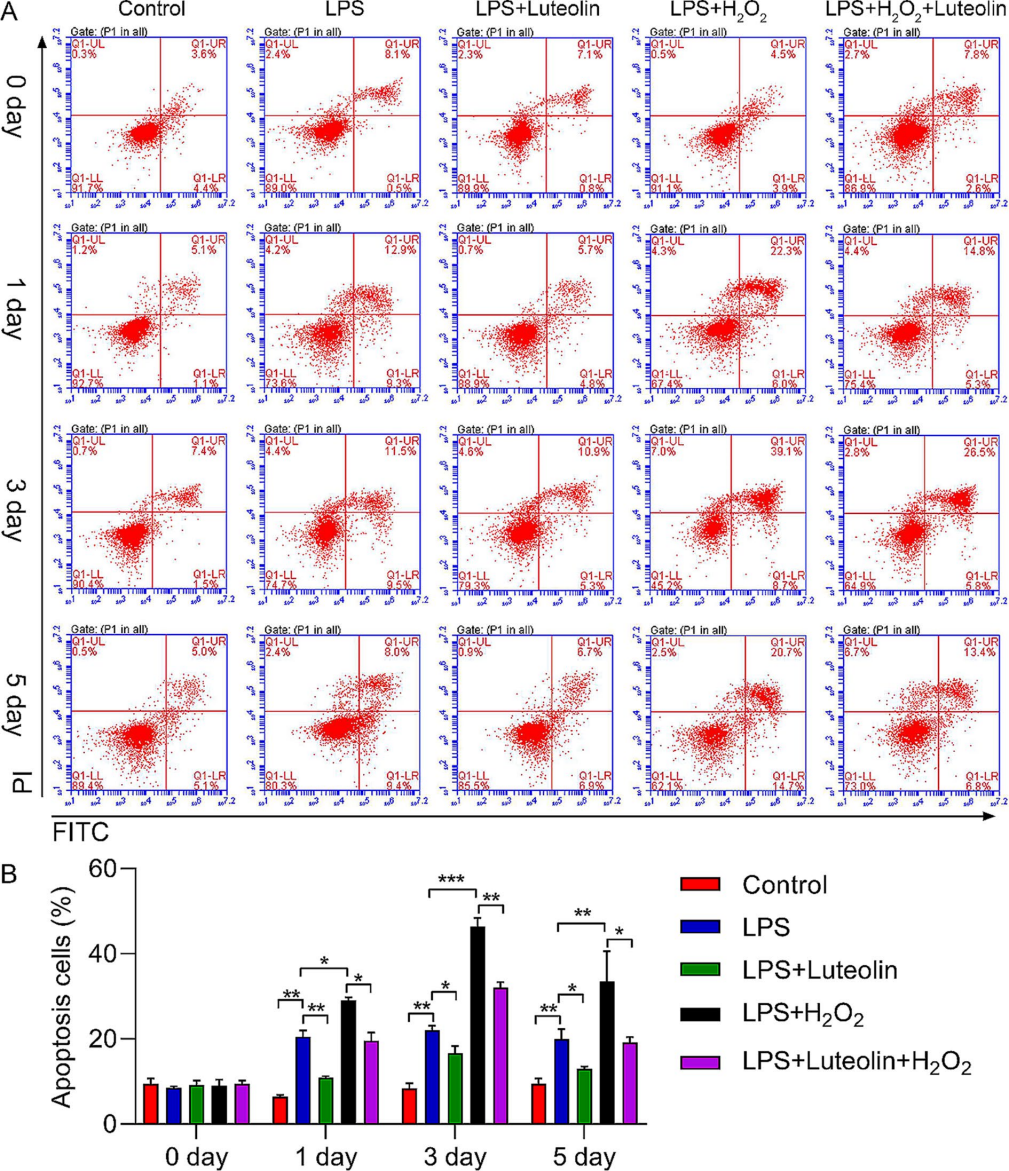
Effect of luteolin on the apoptosis of hFOB1.19 cells. (**A**) Flow cytometry analysis of hFOB1.19 cells. (**B**) Quantitative analysis of apoptotic cells via flow cytometry analysis. LPS-induced cell apoptosis increased in hFOB1.19 cells after 1, 3, and 5 days, and this increase was reversed by luteolin. With the combination of LPS and H_2_O_2_, cell apoptosis significantly increased in hFOB1.19 cells after 1, 3, and 5 days. This effect was also alleviated by luteolin. The data are expressed as the means ± SDs (***p < 0.001, **p < 0.01, *p < 0.05)

We subsequently tested cell apoptosis via flow cytometry analysis. We found that LPS treatment notably promoted the apoptosis of hFOB1.19 cells. In addition, H_2_O_2_ treatment markedly increased the percentage of apoptotic hFOB1.19 cells compared with that in the LPS-treated group. Luteolin treatment reduced cell apoptosis, thus confirming the antiapoptotic effect of luteolin. Luteolin also partly rescued the pro-apoptotic effect of H_2_O_2_ on hFOB1.19 cells after LPS treatment (Fig. 3).

**Fig. 4:**
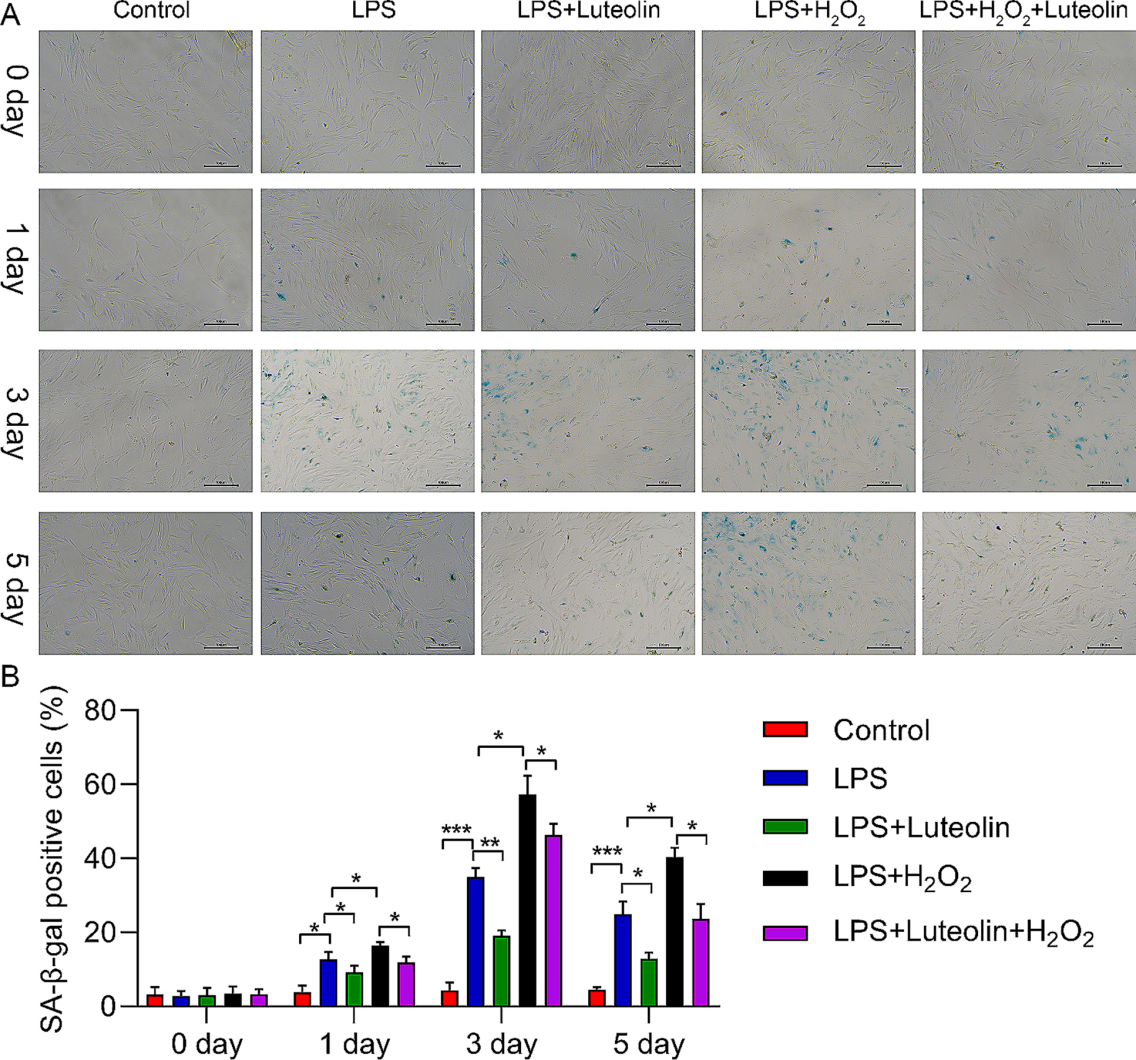
Effect of Luteolin on the senescence of hFOB1.19 cells. (**A**) β-galactosidase staining for the senescence assay. (**B**) Quantitative analysis of β-galactosidase (SA-β-Gal)-positive cells. The SA-β-Gal results demonstrated that hFOB1.19 cells in the combined LPS + H2O2 group and LPS group showed intense blue staining at 1, 3, and 5 d, whereas the staining intensity in the combined LPS + H2O2 group was greater than that in the LPS only group. After being exposed to luteolin for 1, 3, or 5 days, hFOB1.19 cells exhibited weak blue staining, which was not as intense as that in the LPS group or the combined LPS + H2O2 group. The data are expressed as the means ± SDs (***p < 0.001, **p < 0.01, *p < 0.05)

Moreover, we detected cell senescence by using beta-galactosidase (SA-β-Gal) staining. As shown in Fig. 4, LPS treatment promoted cell senescence, and H_2_O_2_ treatment further increased apoptotic cell senescence compared with that in the LPS-treated group. We detected that luteolin inhibited cell senescence, which also confirmed the cytoprotective effect of luteolin treatment. Luteolin partly rescued the effect of H_2_O_2_ on osteoblast cell senescence under LPS treatment (Fig. 4). Therefore, these results indicated that luteolin reduced H_2_O_2_-induced cell injury.

### Luteolin ameliorated oxidative stress induced by H_2_O_2_ in vitro

**Fig. 5:**
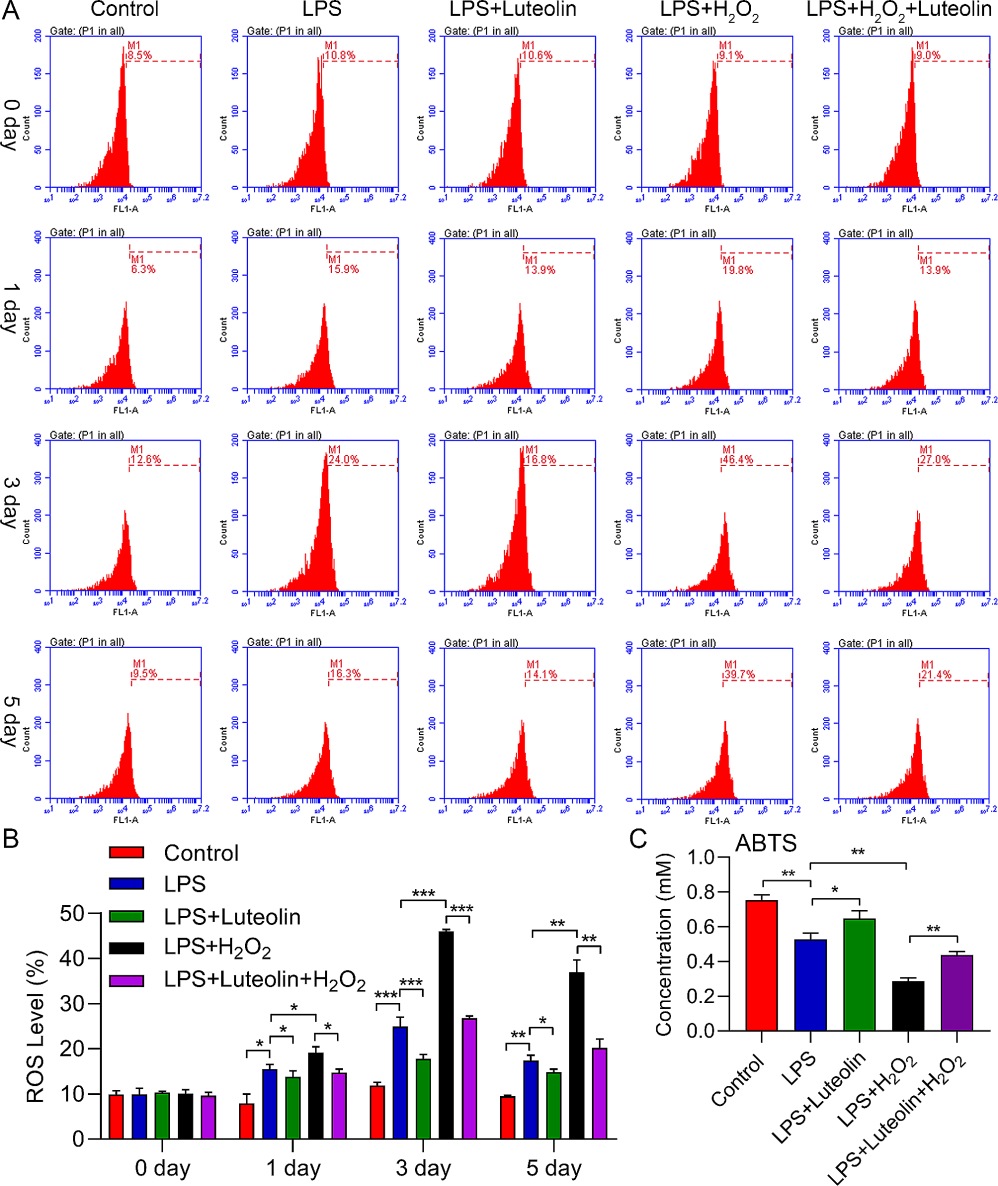
Effect of LPS on ROS production in hFOB1.19 cells. (**A**) Flow cytometry analysis of hFOB1.19 cells. (**B**) Quantitative ROS analysis via flow cytometry. ROS increased in hFOB1.19 cells after 1, 3, and 5 days of LPS induction, and this increase was reversed by luteolin. Combined with LPS and H_2_O_2_ induction, ROS were significantly increased in hFOB1.19 cells after 1, 3, and 5 days. This effect was also alleviated by luteolin. (**C**) 2,2′-azino-bis(3-ethylbenzothiazoline-6-sulfonic acid) (ABTS) free radical scavenging assay of hFOB1.19 cells after 3 days. ABTS decreased in hFOB1.19 cells after 1, 3, and 5 days of LPS induction, and this decrease was reversed by luteolin. Combined with LPS and H_2_O_2_ induction, ROS were significantly decreased in hFOB1.19 cells after 3 days. This effect was also alleviated by luteolin. The data are expressed as the means ± SDs (***p < 0.001, **p < 0.01, *p < 0.05)

Due to the fact that inflammation is associated with oxidative stress [17, 18], the intracellular ROS and ABTS levels were tested. ROS levels were significantly greater in the LPS-treated group than in the control group. Furthermore, H_2_O_2_ treatment further notably increased the ROS level compared to that in the LPS-treated group, which was opposite to what was observed in the luteolin-treated group. Additionally, luteolin partly rescued the effect of H_2_O_2_ on ROS production after LPS treatment (Fig. 5A, B). As shown in Fig. 5C, the change in the ABTS level was opposite to that in the ROS level (Fig. 5C). These results indicated that luteolin ameliorated oxidative stress induced by H_2_O_2_ in vitro.

### Luteolin alleviated H_2_O_2_-induced upregulated expression of proinflammatory cytokines

**Fig. 6:**
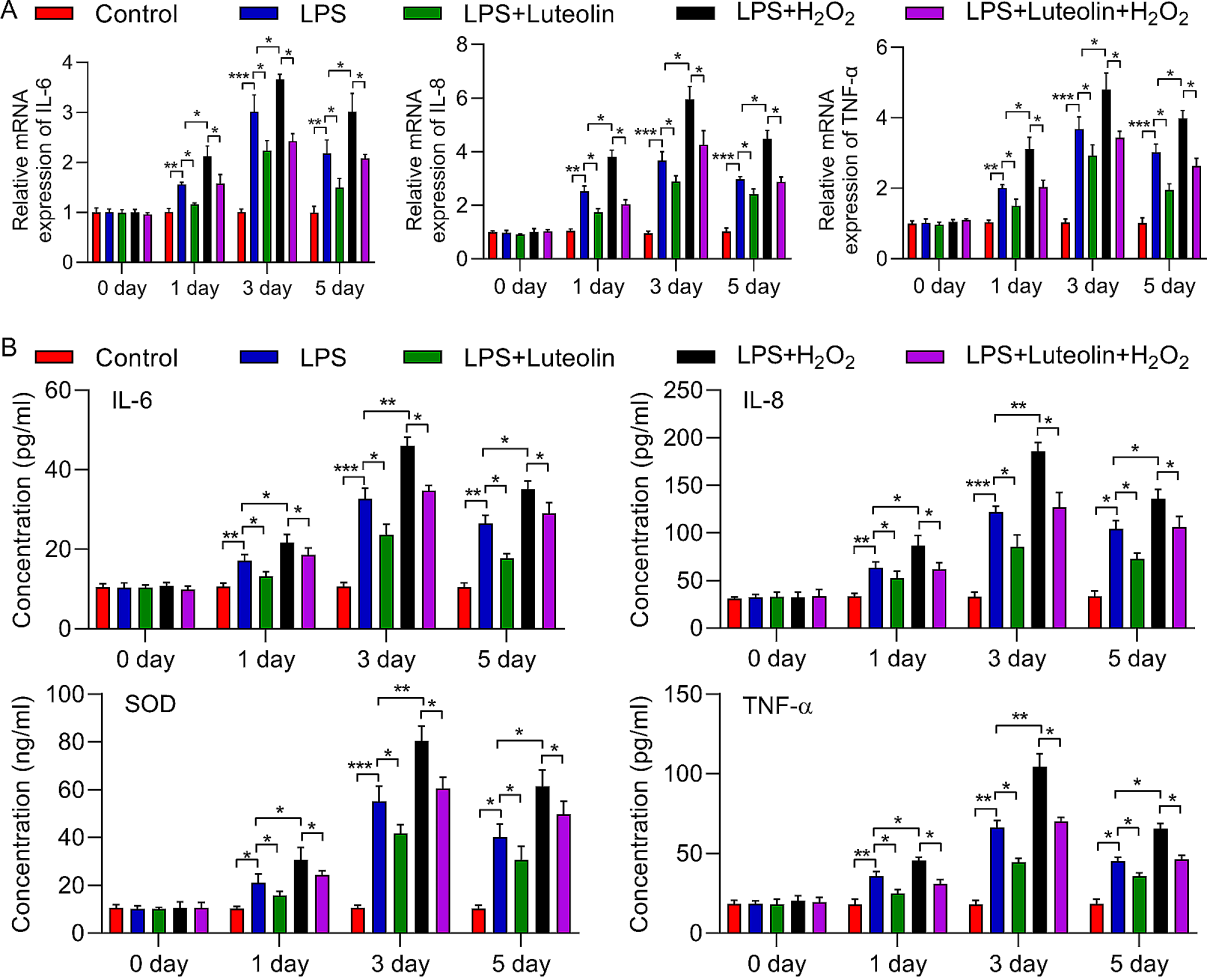
Effect of luteolin on the mRNA expression of hFOB1.19 cells and its downstream targets. (**A**) The relative expression levels of IL-6, IL-8 and TNF-α were determined via RT‒qPCR. The mRNA (**A**) and protein (**B**) levels of IL-6, IL-8 and TNF-α were increased in hFOB1.19 cells after 1, 3, and 5 days of LPS induction, and this increase was moderated by luteolin. With LPS combined with H_2_O_2_, IL-6, IL-8 and TNF-α expression significantly increased in hFOB1.19 cells after 1, 3, and 5 days. This effect was also alleviated by luteolin. The protein level (**B**) of SOD decreased in hFOB1.19 cells after 1, 3, and 5 days of LPS induction, and this decrease was reversed by luteolin. With the combination of LPS and H_2_O_2_, IL-6, IL-8 and TNF-α expression significantly decreased in hFOB1.19 cells after 1, 3, and 5 days. This effect was also alleviated by luteolin. The data are expressed as the means ± SDs (***p < 0.001, **p < 0.01, *p < 0.05)

To further investigate the effect of luteolin on cell protection, we evaluated the expression and production of proinflammatory cytokines. Remarkable upregulation of IL-6, IL-8 and TNF-α at both the mRNA and protein levels was observed in hFOB1.19 cells treated with LPS. In addition, compared with LPS treatment, H_2_O_2_ treatment further increased the expression of these proinflammatory cytokines. In contrast, luteolin significantly decreased the expression of IL-6, IL-8 and TNF-α, which was increased by LPS treatment. Additionally, luteolin partly rescued the effect of H_2_O_2_ on proinflammatory cytokine expression after LPS treatment (Fig. 6A, B). Therefore, luteolin inhibited the production of proinflammatory cytokines induced by H_2_O_2_.

### Luteolin inhibited the expression of inflammation-associated proteins in H_2_O_2_-induced cell injury

**Fig. 7:**
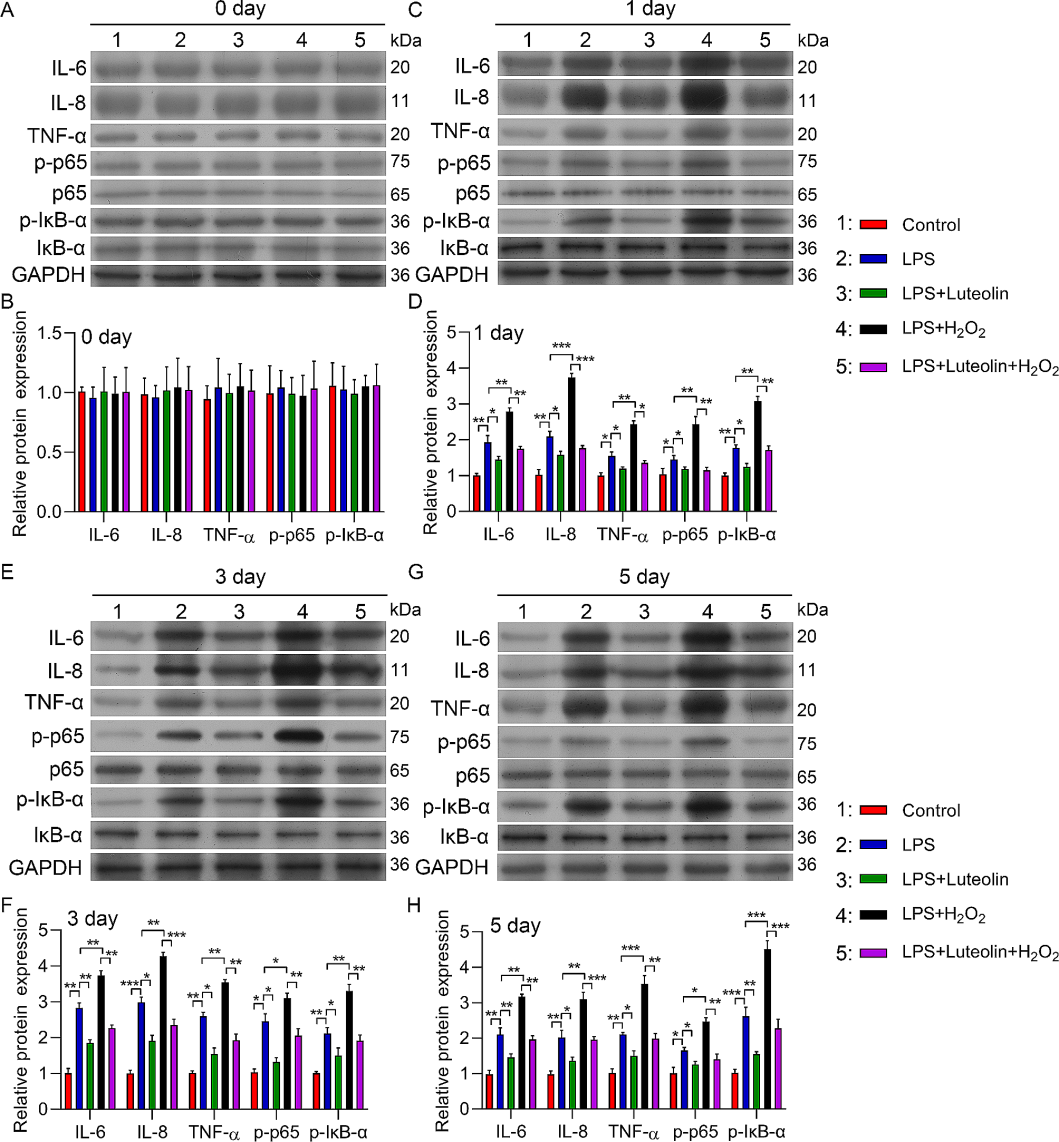
Effect of luteolin on the protein expression of hFOB1.19 cells and its downstream targets. (**A**, **C**, **E**, **G**) Western blotting analysis of IL-6, IL-8, TNF-α, p-p65 and p-lκB-α. (**B**, **D**, **F**, **H**) Relative protein expression levels of IL-6, IL-8, TNF-α, p-p65, p65, lκB-α, and p-lκB-α. The protein levels of IL-6, IL-8, TNF-α, p-p65, and p-lκB-α were increased in human osteoblasts after 1, 3, and 5 days of LPS induction, and this increase was moderated by luteolin. Combined with LPS and H_2_O_2_ induction, IL-6, IL-8, and TNF-α expression significantly increased in human osteoblasts after 1, 3, and 5 days. This effect was also alleviated by luteolin. The data are expressed as the means ± SDs (***p < 0.001, **p < 0.01, *p < 0.05)

The NF-κB signalling pathway is closely related to inflammation [19, 20]. Therefore, to further elucidate the underlying mechanism of the protective effect of luteolin, we examined the classic inflammatory NF-κB signalling pathway via western blotting analysis. Compared with those in the control group, IL-6, IL-8, TNF-α, p-p65, and p-lκB-α expression was notably increased; additionally, Nrf2 expression was decreased in hFOB1.19 cells treated with LPS, and luteolin significantly decreased IL-6, IL-8, TNF-α, p-p65, and p-lκB-α expression and increased Nrf2 expression in LPS-induced hFOB1.19 cells. Furthermore, H_2_O_2_ treatment further upregulated IL-6, IL-8, TNF-α, p-p65, and p-lκB-α and downregulated Nrf2 in LPS-induced hFOB1.19 cells; moreover, luteolin partly rescued H_2_O_2_-mediated regulation of IL-6, IL-8, TNF-α, p-p65, p-lκB-α, and Nrf2 expression in LPS-induced hFOB1.19 cells (Fig. 7A–H). Overall, luteolin may inhibit H_2_O_2_-induced cell injury by inhibiting the NF-κB signalling pathway.

### Luteolin upregulates Nrf2 expression in H_2_O_2_-induced cell injury

**Fig. 8:**
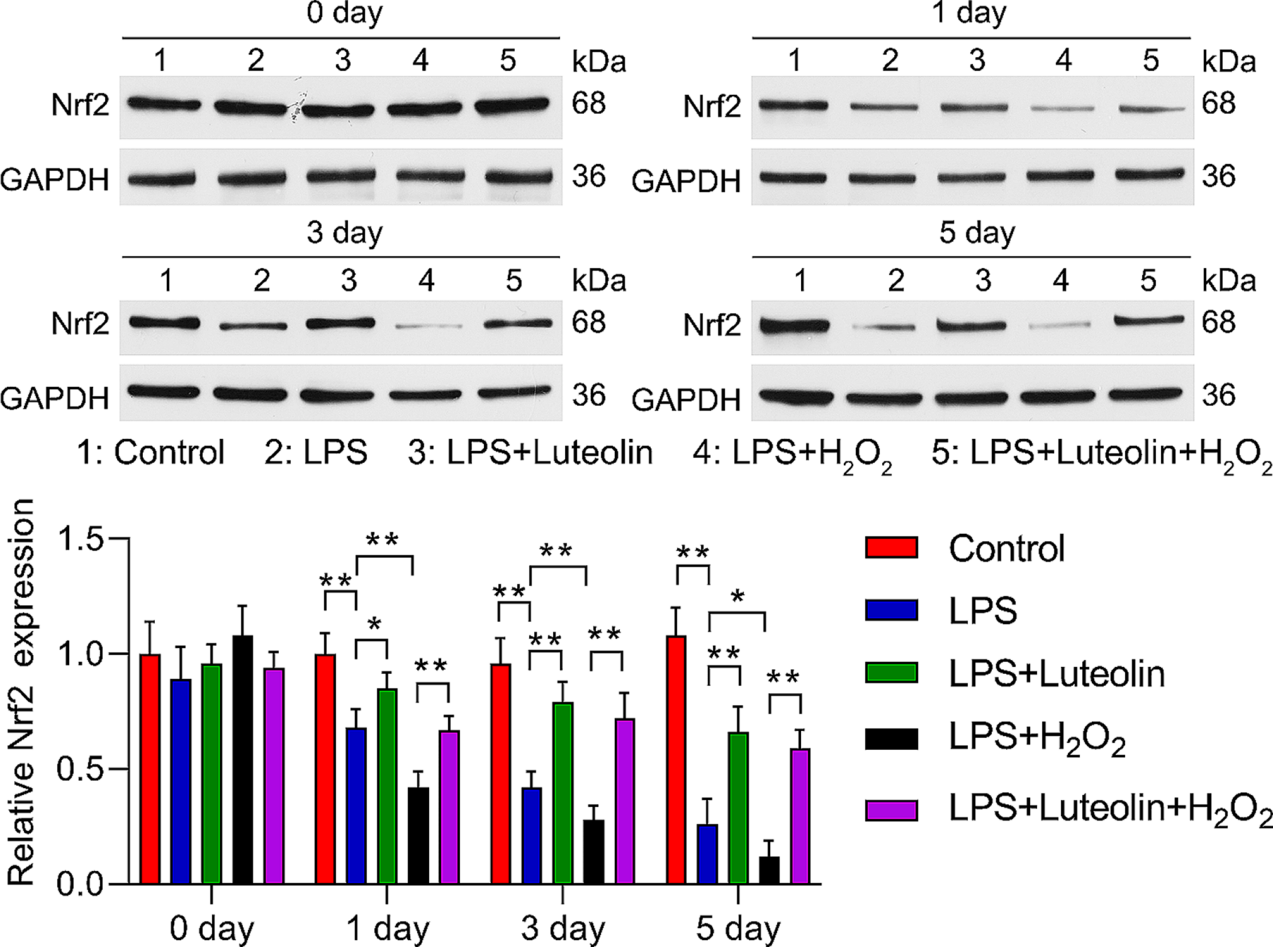
Effect of luteolin on Nrf2 expression in hFOB1.19 cells. Western blotting analysis of Nrf2 expression in treated hFOB1.19 cells. Nrf2 levels decreased in hFOB1.19 cells after 1, 3, and 5 days of LPS induction, and this effect was reversed by luteolin. After LPS and H_2_O_2_ induction, Nrf2 expression decreased in hFOB1.19 cells after 1, 3, and 5 days. This effect was also alleviated by luteolin. The data are expressed as the means ± SDs (**p < 0.01, *p < 0.05)

In addition, the Western blotting results demonstrated that compared with that in the control group, Nrf2 expression was decreased in the hFOB1.19 cells treated with LPS. Additionally, luteolin markedly increased Nrf2 expression in the LPS-induced hFOB1.19 cells, H_2_O_2_ treatment downregulated Nrf2 in the LPS-induced hFOB1.19 cells, and luteolin reversed the regulatory effect of H_2_O_2_ on Nrf2 expression in the LPS-induced hFOB1.19 cells (Fig. 8).

## Discussion

A periapical lesion can eventually lead to severe bone resorption and tooth loss [21]. Periapical lesions are infectious diseases that occur in the apical region of teeth. The development and progression of periapical lesions are dependent on interactions between pathogens and the host immune system. Therefore, anti-infection therapy is crucial for patients. H_2_O_2_ is widely used in clinical practice. However, H_2_O_2_ causes inflammation and damages osteoblasts. Anti-inflammatory therapy is necessary during H_2_O_2_ treatment. The aim of this study was to demonstrate the protective effect of luteolin on osteoblasts. Research has shown that LPS can induce inflammation and osteoclast bone resorption [22]. Based on previous studies [23–25], this study also used LPS to stimulate hFOB1.19 cells to establish an inflammatory model. In addition, we further investigated the role of luteolin in H_2_O_2_-induced oxidative stress in LPS-induced human osteoblasts (hFOB1.19 cells). We further investigated its antioxidative and anti-inflammatory effects and explored the underlying mechanism, which may provide a potential drug for H_2_O_2_-induced periodontitis and osteoblast injury.

More than 5000 flavonoids have been identified and are distributed in a wide range of plants. There were 10 categories based on their chemical structures. Interestingly, 6 categories, including flavonoids, flavanones, anthocyanidins, flavonols, isoflavones, and catechins, are commonly present in the human diet. Many flavonoids possess powerful anticancer activity both in vivo and in vitro [26]. Flavonoids, including luteolin, have been reported to play important roles in potent anticancer effects in several models [27, 28]. Luteolin has been reported to regulate several biological processes, such as apoptosis [29], inflammation [30], ferroptosis [31], and autophagy [32]. Luteolin can suppress the progression of breast cancer and induce cancer cell apoptosis through its antioxidant activity and is a potential anticancer drug for future applications [33]. Luteolin has been reported to protect cells from sevoflurane-induced neurotoxicity by regulating HMOX1, thus activating the autophagy pathway and inhibiting the inflammatory response [34]. Luteolin is associated with the progression of nonalcoholic fatty liver disease by suppressing the TLR4/NF-κB pathway to change gut bacterial species [35]. These studies have indicated that luteolin may be a powerful potential drug for treating several diseases, including nonalcoholic fatty liver disease, cancer and infection. Luteolin has been reported to protect osteoblasts from antimycin A-induced injury, methylglyoxal-induced injury and glucose-induced injury [36–38]. These results were consistent with our present findings. We detected that luteolin protects osteoblasts from inflammation-induced injury. To our knowledge, this is the first study to associate luteolin- and H_2_O_2_-induced inflammation with osteoblast cell survival.

The relationship between ROS and inflammation has been well explored. Decreased ROS production has been reported to mediate the inhibitory effect of aloin on LPS-induced inflammation [39]. ROS can effectively regulate pathogenic and inflammatory immune responses and suppress the effects of MDSCs [40]. ROS production has even been reported to promote cancer-associated inflammation and cancer progression [41]. Accumulating studies have reported that ROS promote periodontitis progression [42–46]. Additionally, it has been reported that ROS induced by triggering receptors expressed on myeloid cells 2 regulate osteoclastogenesis in periodontitis [47]. ROS overproduction also mediates ligature-induced periodontitis by regulating the ratio of M1 to M2 macrophages in diabetes [48]. In our present study, higher ROS levels were also detected in cells treated with H_2_O_2_. Moreover, H_2_O_2_ also upregulated the expression of proinflammatory cytokines, such as IL6 and IL8, which ultimately induced cell injury. Therefore, we confirmed that luteolin protects hFOB1.19 cells from inflammation-induced injury by inhibiting ROS production.

Herein, we demonstrated that luteolin regulated ROS and inflammation by inhibiting the NF-κB signalling pathway. NF-κB is well understood to be involved in many molecular processes, such as IL-6, TNF-α, and TLR4, in cancer [49]. When considering inflammation, the NF-κB signalling pathway reportedly increases the expression of inflammatory cytokines (TNF-α) and subsequently promotes inflammation [50]. However, ROS and inflammation can also regulate the NF-κB signalling pathway through the ERK/AKT pathway and form a positive feedback loop [51]. As previously reported, NF-κB signalling pathway activation promoted apical periodontitis progression via crosstalk with the Wnt signalling pathway [52]. The NF-κB signalling pathway also mediates the effects of BCP and PRF on chronic periodontitis [53]. Therefore, our findings were consistent with those of previous studies. In addition, luteolin inhibited NF-κB-mediated inflammation and activated Nrf2-mediated antioxidant responses to protect against diabetic cardiomyopathy and neuronal cell injury [54–56]. These findings indicated that the NF-κB pathway may be the main downstream target involved in regulating ROS and inflammation.

There were several limitations in our study. First, we demonstrated that luteolin regulated the NF-κB signalling pathway in vitro. Animal experiments are needed to verify the effects of luteolin. Second, the current study only used the hFOB1.19 cell line to explore the possible role and mechanism of luteolin; thus, to increase the reliability of our results, we should verify whether luteolin has similar effects on other human osteoblasts in future studies. Third, the physicochemical state of the cell membrane may affect the ability of luteolin to protect against H_2_O_2_-induced cell damage in osteoblasts. In future studies, it will be crucial to further explore the relationship between cell membrane disruption or stabilization and the protective effect of luteolin on osteoblasts. Finally, the effects of luteolin on osteoblast cell protection may not be solely dependent on the NF-κB signalling pathway. Luteolin can stimulate the mineralization of human osteoblasts via the Wnt pathway [57]; moreover, luteolin can downregulate MMP-9 and MMP-13 expression in osteoblasts via the ERK pathway [58], as well as suppress epithelial–mesenchymal transition and subretinal fibrosis via the Smad2/3 and YAP pathways [59]. The regulation of these molecules and pathways by luteolin should also be verified in further studies.

## Conclusions

We demonstrated that luteolin protected hFOB1.19 cells from H_2_O_2_-induced cell injury and inhibited the expression of proinflammatory cytokines by inhibiting the NF-κB signalling pathway. Our findings provide a potential drug for treating H_2_O_2_-induced periodontitis and osteoblast injury.

## Data Availability

The datasets used and/or analysed during the current study are available from the corresponding author upon reasonable request.

## Abbreviations

LPS: Lipopolysaccharide
ROS: Reactive oxygen species
RT‒qPCR: Reverse transcription quantitative polymerase chain reaction
ELISA: Enzyme-linked immunosorbent assay
NF-κB: Nuclear factor kappa-B
TNF-α: Tumour necrosis factorα
IL-6: Interleukin 6
IL-8: Interleukin 8
IL-2: Interleukin-2
CRP: C-reactive protein
SOD: Superoxide dismutase
